# 

**Published:** 1903-10-15

**Authors:** 


### ANNOUNCEMENTS.

THE Seventh and Eighth District Dental Societies, of New York, will meet October 27th, 28th and 29th, at the Osborn House in Rochester, N. Y. One day will be devoted exclusively to clinics, the evening being given up to a discussion of the clinics. Thirty-six clinics and seven papers are already promised. This ought to be a good meeting.

THE Board of Dental Examiners of the State of Ohio will meet in Columbus, Ohio, November 24th, 25th and 26th, at the Hotel Hartman, for examination of candidates for certificates of registration. Application should be filed with the Secretary by November 14th. For further particulars address H. C. Brown, Secretary, 185 E. State Street.

NATIONAL ASSOCIATION OF DENTAL EXAMINERS.—It is earnestly requested that all the secretaries of the Boards of Examiners throughout the States and Territories mail to the Secretary all changes in their respective Boards and greatly oblige. Chas. A. Meeker, D.S., Secretary, 29 Fulton Street, Newark, N. J.

THE Fraternal Dental Society of St. Louis, by special resolutions, endorses the International Dental Congress, to be held in St. Louis August 29th to September 3d, 1904, and promises to co-operate as a body in any way it can to secure the success of the Congress.

THE next annual meeting of the Institute of Dental Pedagogics will be held at Buffalo, N. Y., December 28th,

29th and 30th. A complete program is being arranged which will be exceedingly interesting. It will be published in full in the next issue of this journal.

On account of the resignation of Burton L. Thorpe, D.D.S., President of the National Association of Dental Examiners, James G. Reid, D.D.S., of 1204 Trude Building, Chicago, Ills., will assume the duties of the president. Chas. A. Meeker, D.D.S., Secretary.

Dr. E. B. Lodge, whose office in Cleveland was destroyed by fire last spring has resumed practice with offices in the Rose Building, Cleveland.

